# Toxic Effects of Trazodone on Male Reproductive System via Disrupting Hypothalamic-Pituitary-Testicular Axis and Inducing Testicular Oxidative Stress

**DOI:** 10.1155/2018/7196142

**Published:** 2018-07-29

**Authors:** Sinem Ilgın, Gözde Aydoğan-Kılıç, Merve Baysal, Volkan Kılıç, Mina Ardıç, Şeyda Uçarcan, Özlem Atlı

**Affiliations:** ^1^Faculty of Pharmacy, Department of Pharmaceutical Toxicology, Anadolu University, Eskisehir, Turkey; ^2^Faculty of Science, Department of Biology, Anadolu University, Eskisehir, Turkey

## Abstract

Depression and anxiety are recognized as public health problems. Epidemiological studies have shown that depression and anxiety often occur during reproductive ages between 20 and 60 years of age in males. Trazodone is one of the most frequently prescribed drugs in the treatment of depression and anxiety. Drugs used in repeated doses also play a role in the etiology of infertility. In our study, it was aimed to identify the possible toxic effects of trazodone on male rats and elucidate the underlying mechanisms. Vehicle or trazodone (5, 10, and 20 mg/kg/day) was administered to rats for 28 consecutive days (*n* = 8 per group). At the end of that period, sperm concentration, motility, morphology, and DNA damage were determined and testicular morphology was assessed histopathologically in rats. Additionally, we investigated hormonal status by determining serum testosterone, FSH, and LH levels and oxidative stress by determining glutathione and malondialdehyde levels in testicular tissue to elucidate mechanisms of possible reproductive toxicity. According to our results, sperm concentration, sperm motility, and normal sperm morphology were decreased; sperm DNA damage was increased in trazodone-administered groups. Degenerative findings on the testicular structure were observed after trazodone administration in rats. Additionally, serum FSH, LH, and testosterone levels were elevated in the trazodone-administered groups. Increased MDA levels were the signs of enhanced oxidative stress after trazodone administration in testis tissues. Thus, we concluded that trazodone induced reproductive toxicity in male rats; this reproductive toxicity was accompanied by oxidative stress and hormonal changes, which are considered as important causes of reproductive disorders.

## 1. Introduction

Reproductive health affects the quality of life of the individual as well as both maternal and fetal health during pregnancy, newborn, infant, and child health after pregnancy. In this respect, reproductive health has become an important research area in recent years [1, 2]. Infertility, which is defined as the failure to achieve a clinical pregnancy after 12 months or more of regular unprotected sexual intercourse, is an important disease of the reproductive system. Although it is considered as a widespread health problem worldwide, its incidence and prevalence is very difficult to determine [2–4]. At least 30 million people are stated to be infertile worldwide. It is even more difficult to determine the incidence of male infertility due to cultural constraints [3]. The etiology of infertility arises from female-derived factors, male-derived factors, and both of female- and male-derived factors, which are 40%–50%, 30%, and 20% of all cases, respectively [5, 6]. It could be said that direct or indirect male-derived infertility constitutes approximately 30–50% of the infertility cases [3, 7].

In the etiology of the male infertility, several factors such as varicocele, accessory gland infection, immunological factors, malignancies, genetic abnormalities, endocrine disturbances, and congenital abnormalities have been reported [8–10]. However, idiopathic causes account for 30–40% of male infertility [10, 11]. Obesity, radiation, climate, environmental factors, occupation, and various chemical agents such as pesticides, environmental pollutants, industrial products, and drugs may play a role in the etiology of male infertility [12–15]. The effects of many drugs on male reproductive system have been evaluated in numerous studies. Particularly, drug exposure in repeated doses causes infertility by affecting sperm parameters such as sperm count, motility, and morphology, hypothalamic-hypophyseal axis, Sertoli/Leydig/germ cells in testicular structure, and auxiliary sex organs [16–19].

Depression is a common disease affecting about 300 million people worldwide [20]. Selective serotonin reuptake inhibitors (SSRIs) such as sertraline, fluoxetine, citalopram, escitalopram, paroxetine, fluvoxamine, and trazodone (TRZ) are often prescribed for the treatment of depression due to their high efficacy, safety, and tolerability. SSRIs are known to cause sexual adverse effects (erectile dysfunction, decreased libido, and anorgasmia) [21]. It is also known that serotonin plays a role in reproductive hormonal regulation and spermatogenesis [22, 23]. Some studies have indicated relationship between infertility and hyperserotonemia [22]. Additionally, clinically studies, between SSRI and sperm parameters, have also been reported that sperm count, sperm motility, and normal sperm morphology were decreased in patients who were undergoing SSRI treatment [24–31].

TRZ is an antidepressant of the serotonin antagonist and reuptake inhibitor class. TRZ also has anxiolytic and sleep-inducing effects [32, 33]. It is the most prescribed drug for the treatment of insomnia [34]. Otherwise, it is noteworthy that there has been no published study on reproductive toxicity of TRZ which is frequently used during reproductive ages in males. Therefore, in this study, it was aimed to investigate male reproductive toxicity by determining the sperm concentration, motility and morphology, DNA damage, and histopathologic examination of testis tissues after TRZ exposure at repeated pharmacological doses in rats. In addition, a possible mechanism of action of the adverse reproductive effects was evaluated via the determination of the oxidative status of testis tissue and serum testosterone and follicle-stimulating hormone (FSH) and luteinizing hormone (LH) levels.

## 2. Materials and Methods

### 2.1. Materials

Desyrel® (50 mg tablet, Angelini Pharma) was used for experimental studies. Testosterone, FSH, LH, MDA, and GSH levels were determined using ELISA kits from Cusabio Biotech Co. Ltd. (Wuhan, China).

### 2.2. Animal Model and Experimental Design

Male Sprague-Dawley rats (12 weeks old, 200–250 g) were obtained from Anadolu University Research Center for Animal Experiments. The rats were housed in a room with controlled temperature (24°C) with a 12 h light/12 h dark cycle (lights on at 08 : 00 h) with free access to standard rat food and water. Temperature, sound, and light conditions of the laboratory were maintained during the course of the experiments. Animals were acclimatized to the laboratory environment for at least 48 h before experimentation. The experimental protocol was approved by the Local Ethical Committee on Animal Experimentation of Anadolu University, Eskisehir, Turkey (file registration number 2015-08). The rats were assigned randomly into the following treatment groups:

- Control group: animals received distilled water orally for 28 days (*n* = 8).
- 5 mg/kg TRZ-treated group: animals received 5 mg/kg dose TRZ orally for 28 days (*n* = 8).
- 10 mg/kg TRZ-treated group: animals received 10 mg/kg dose TRZ orally for 28 days (*n* = 8).
- 20 mg/kg TRZ-treated group: animals received 20 mg/kg dose TRZ orally for 28 days (*n* = 8).

Pharmacological doses, which were determined in previous studies, were chosen as the study doses of TRZ [35–40]. Furthermore, the clinical dose of TRZ for the treatment of depression is 250–600 mg per day and the proper dose for the treatment of insomnia is 25–100 mg before sleep [41]. The doses we have chosen were in accordance with the guidelines extrapolating human doses to animal doses [42]. All drugs were administered at a volume of 1 ml/100 g by dissolving in distilled water. Concentrations were adjusted to deliver the intended dose levels of the base compound. The treatment period was in accordance with the guideline OECD 407: repeated dose oral toxicity study in rodents [43]. Additionally, 28-day period is considered as suitable for determining xenobiotic-induced reproductive toxic effects in male rats [44].

At the end of 28 days, the animals were anesthetized by intraperitoneal injection of 1.5 mg/kg urethane [45]. Blood samples for hormonal analysis (FSH, LH, and testosterone) were collected from the right ventricle of the animals via syringe. The animals were euthanatized via withdrawal of large amounts of blood from the heart.

Testis and epididymis tissues were removed. The left testis and epididymis were cleaned of blood in phosphate-buffered solution (PBS) (8 g/l NaCl, 0.2 g/l KCl, 0.2 g/l KH_2_PO_4_, 1.14 g/l Na_2_HPO_4_, pH 7.4) and weighed. The left epididymis was used to determine the levels of GSH and MDA. The right testis was cleared of blood and other contaminants in PBS and fixed for histological examination. The cauda of the right epididymis was used to evaluate sperm parameters [46–48].

### 2.3. Collection and Evaluation of Sperm Samples

Spermatozoa obtained immediately after euthanizing the rats from the right epididymis, which was placed in a Petri dish containing DMEM/Hams F-12 at 37°C. The cauda epididymis was transferred to a new Petri dish with 1 ml of the same medium, and the blood vessels and fat tissue were removed. A section of the cauda epididymis (0.5 cm) was cut out and placed in another Petri dish containing 1 ml of the same medium, and spermatozoa were allowed to swim out for 1 min to obtain a cloud of spermatozoa [46–50].

### 2.4. Assessment of Sperm Concentration and Motility

Five microliters of concentrated spermatozoa cloud was collected and placed on a Leja slide (Leja Products BV, Nieuw Vennep, Netherlands). The Leja slide was placed onto a temperature-controlled stage of the Nikon E200 microscope (37°C). A 4x negative phase-contrast objective in conjunction with a phase-contrast condenser was used to determine sperm motility and concentration via the motility/concentration module of the Sperm Class Analyzer® version 5.4.0.1 software (Microptic SL, Barcelona, Spain) at 50 frames/s. Data were collected by capturing images with a digital camera (Basler, A78075gc, Germany). For motility analysis, eight fields were captured with the SCA system until 200 motile spermatozoa were analyzed, as recommended by WHO (1999) [46–48, 51].

### 2.5. Assessment of Sperm Morphology

**Figure 1:**
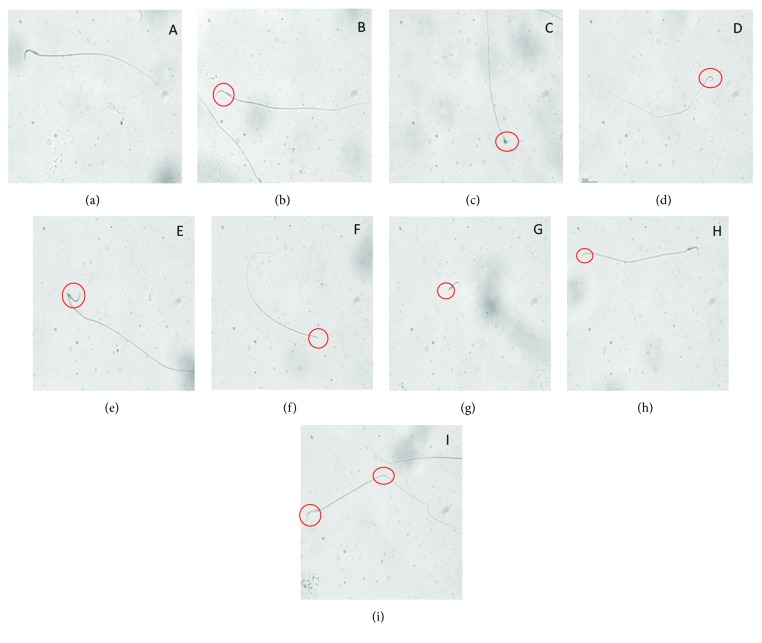
Classification of sperm morphology in rats: (a) normal sperm, (b) banana-shaped head, (c) amorphous head, (d) bent neck, (e) bent neck, (f) headless, (g) tailless, (h) broken tail, and (i) banana-shaped head and bent tail (multiple anomalies).

Fresh sperm smears were prepared for morphometric analysis by placing 5 *μ*l of the fresh semen on the clear end of a frosted slide by dragging the drop across the slide. The smears were air-dried before staining. Three semen smears were prepared and stained with Spermblue® (Microptic Automatic Diagnostic System, Barcelona, Spain) according to Van der Horst and Maree (2009) [52]. Stained slides were used to perform morphology evaluation using the morphometry module of the Sperm Class Analyzer version 5.4.0.1 software (Microptic SL, Barcelona, Spain). The machine was equipped with a Nikon Eclipse model 50i (Nikon Corporation, Tokyo, Japan) microscope with a 60x bright-field objective and a video camera (Basler, A78075gc, Germany). A total of 200 sperms/animal were analyzed. The morphometric parameters of head and tail were determined, and abnormal sperms were detected based on previous criteria [46–48, 53–57]. Sperms with banana-shaped head, amorphous head, bent neck, or two-headed and headless sperms were classified as sperms with head abnormalities, whereas sperms with a bent or broken tail were classified as sperms with tail abnormalities (Figure 1).

### 2.6. Detection of Sperm DNA Damage by Using Comet Assay

Frosted microscope slides were covered with 1% normal melting point agarose in Ca^2+^- and Mg^2+^-free PBS. A sperm sample (10 *μ*l) containing 1 × 10^5^ sperm/ml was suspended in 75 *μ*l of 1% (*w*/*v*) low melting point agarose. This suspension (85 *μ*l) was placed on the surface of a microscope slide (precoated with 1% normal melting point agarose) to form a microgel and allowed to set at 4°C for 5 min. Slides were dipped in cell lysis buffer (2.5 M NaCl, 100 mM EDTA, 10 mM Tris-HCl, pH 10.0, containing 1% Triton X-100 added just before use and 40 mM dithiothreitol) for 24 h at 20 to 22°C. Following the initial lysis, proteinase K was added to the lysis solution (0.5 mg/ml) and additional lysis was performed at 37°C for 24 h. After cell lysis, all slides were washed three times with deionized water at 10 min intervals to remove the salt and detergent from the microgels. Slides were placed in a horizontal electrophoresis unit and were allowed to equilibrate for 20 min with running buffer (500 mM NaCl, 100 mM Tris-HCl, 1 mM EDTA, pH 9) before electrophoresis (0.60 V/cm, 250 mA) for 30 min. After electrophoresis, slides were then neutralized with 0.4 M Tris (pH 7.5), stained with SYBR Green I (1 : 10,000) for 1 h and covered with cover slips. Slides were analyzed using Leica DM1000 fluorescence microscope (Leica Microsystems, Wetzlar, Germany) and Comet Assay IV Windows XP Pro software (Perceptive Instruments, Suffolk, United Kingdom). At least 100 cells were analyzed per sample [46–48, 58].

### 2.7. Histological Analysis of Testis Tissue

The right testis tissues were sliced into small pieces (2 mm^3^) and then fixed in paraformaldehyde (4%) in phosphate buffer pH 7.2 for 2 h at 20 to 22°C. They were dehydrated in a graded series of alcohols. In order to improve infiltration, the samples were treated with a mixture of LR White (Electron Microscopy Sciences, FT Washington, PA) and 70% ethanol (2 : 1) (*v*: *v*) for 1 h at 20 to 22°C. The samples were then embedded in LR White and sectioned at 700 nm (0.7 microns) thickness by using a Leica EM UC7 ultramicrotome. Semithin sections were stained with 1% toluidine blue/borax (pH 8.4) for 2 min and observed under a Leica DM 750 light microscope [46–48, 59]. Spermatogenesis and testicular injury were evaluated using Johnsen's mean testicular biopsy score criteria. A score of 1–10 was assigned to each tubule cross section (*n* = 160) according to the range from no cells to complete spermatogenesis. Complete spermatogenesis with many spermatozoa present is evaluated as score 10. The total Johnsen score is then determined by dividing the total score by the number of evaluated tubules. At the cellular level, three pathological viewpoints (spermatogonial swelling, cytoplasmic vacuolation, and deformation of cellular architecture) were estimated on a semiquantitative scale and indicated as low (+), moderate (++), and high (+++) according to their degrees [60].

### 2.8. Determination of Serum FSH, LH, and Testosterone Levels

After 30 min of drawing the blood to allow clotting, blood samples from rats were centrifuged at 1000*g* for 15 min at 4°C, and serum was separated. The hormonal analyses were performed using the commercially available kits according to the manufacturer's instructions.

### 2.9. Determination of GSH and MDA Levels in Testis Tissue

The right testis was divided into equal parts and stored at −20°C after freezing in liquid nitrogen. The GSH and MDA levels in the testis were determined by using commercially available kits according to the instructions of the manufacturer.

### 2.10. Statistical Analysis

All data were expressed as mean ± standard error. Statistical analyses of the groups were performed using the SigmaPlot v.10 package program (Systat Software, USA). All values were verified to be normally distributed. For the sperm comet assay, the Dunnett T3 test was performed as a post hoc test. In the other experiments, one-way analysis of variance following the Tukey test as a post hoc test was performed. *p* < 0.05 was considered statistically significant.

## 3. Results

### 3.1. Effects of TRZ Treatment on Testis and Epididymis Weights in Rats

When relative testis and epididymis weights were compared among groups, relative testis and epididymis weights obtained from the TRZ-administered groups were indistinguishable from the control group. No significant differences were observed among the TRZ-administered groups in terms of relative testis and epididymis weights (Table 1).

### 3.2. Effects of TRZ Treatment on Sperm Concentration, Motility, and Morphology in Rats

When the groups were compared in terms of sperm concentration, significant and dose-related decreases in sperm concentration were observed in all TRZ-administered groups compared to the control group. No significant differences were observed among the TRZ-administered groups (Table 2).

Sperm motility percentages of TRZ-administered groups were decreased significantly and dose-dependently when compared to control group. No significant differences were observed among the TRZ-administered groups. (Table 2).

A significant increase in the percentage of the sperm abnormalities was observed in the TRZ-administered groups at all concentrations compared to control. Among the TRZ-administered groups, the percentages of the sperm abnormalities did not show any significant differences (Table 2).

These abnormalities were found to be more in the tail, as represented by bent and broken tail abnormalities (Figure 1), in the 5, 10, and 20 mg/kg TRZ-administered groups at 64.50%, 70.92%, and 78.30%, respectively. The percentages of sperm head abnormalities, including banana-shaped head, amorphous head, bent neck, and two-headed and headless sperms (Figure 1), were determined to be 33.29%, 27.83%, and 20.34% in the 5, 10, and 20 mg/kg TRZ-administered groups, respectively. The percentages of multiple abnormalities were 2.21%, 1.25%, and 1.36% in the 5, 10, and 20 mg/kg TRZ-administered groups, respectively.

### 3.3. Effects of TRZ Treatment on Sperm DNA in Rats

**Figure 2:**
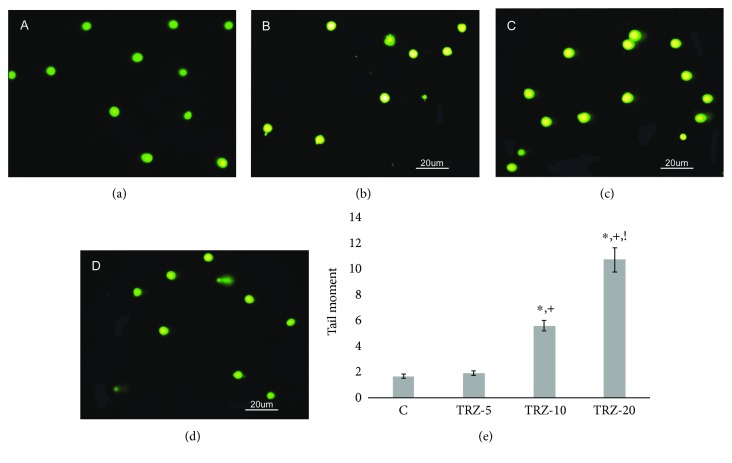
(a–d) DNA damage in rat sperms in control and experimental group animals exposed to physiological saline (0.9%) or different doses of trazodone. (a) Sperm comet assay photo of control rats. (b) Sperm comet assay photo of 5 mg/kg trazodone-administered rats. (c) Sperm comet assay photo of 10 mg/kg trazodone-administered rats. (d) Sperm comet assay photo of 20 mg/kg trazodone-administered rats. (e) Tail moment graph: ^∗^Different from C (*p* < 0.05); ^+^Different from TRZ5 (*p* < 0.05); ^!^Different from TRZ10.

Results of the comet assay are expressed as extent tail moment, a product of the tail length and the tail DNA% (extent tail moment = tail length x tail DNA%/100) (Lee et al., 2004). Values were as the following: 1.65 ± 0.17, 1.91 ± 0.18, 5.55 ± 0.41, and 10.66 ± 0.92 (mean ± SE) for control, 5 mg/kg, 10 mg/kg, and 20 mg/kg TRZ-administered groups, respectively. Control group and 5 mg/kg TRZ-administered group did not show any significant damage. Exposure to 10 and 20 mg/kg TRZ increased the tail moment over 3-fold and 6-fold, respectively, when compared to control (Figure 2).

### 3.4. Effects of TRZ Treatment on the Testicular Histology in Rats

**Figure 3:**
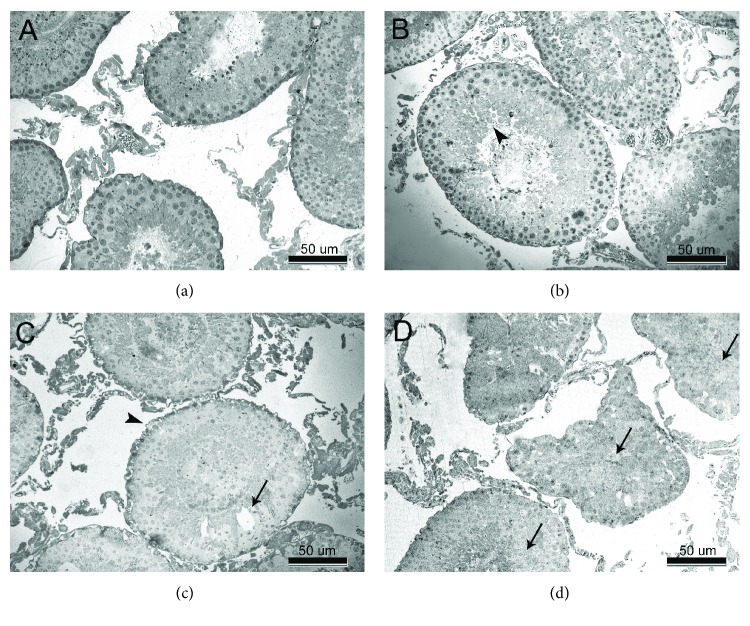
(a–d) Transverse section of seminiferous tubules. (a) Normal appearance of seminiferous tubules in control rats. (b) Mild central degeneration in seminiferous tubules (arrowhead) in 5 mg/kg trazodone-administered rats. (c) Deformation in the basement membrane of seminiferous tubules (arrowhead) and large vacuoles (arrow) in germinal epithelium in 10 mg/kg trazodone-administered rats. (d) Seminiferous tubules with total degeneration (arrow) in 20 mg/kg trazodone-administered rats.

**Figure 4:**
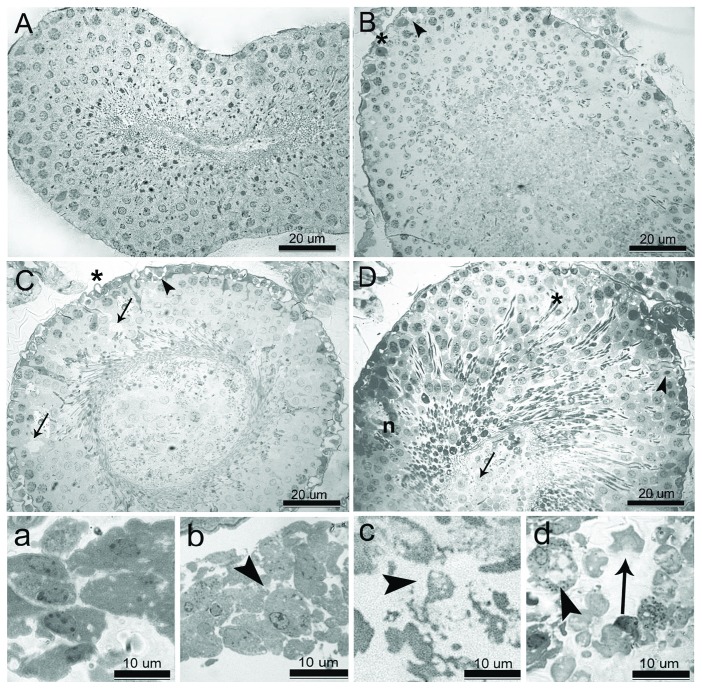
(A–D) High magnification of seminiferous tubules. (A) Intact structure of germinal epithelium in control rats. (B) Irregularity of basement membrane (^∗^) and vacuolation in Sertoli cells (arrowhead) in 5 mg/kg trazodone-administered rats. (C) Detachment of basement membrane (^∗^), deformation in Sertoli cells (arrowhead), and vacuolar degeneration (arrow) in germinal epithelium 10 mg/kg trazodone-administered rats. (D) Disintegration of germinal epithelium (^∗^), desquamation of cells into the lumen (arrow), and necrosis in Sertoli cells and neighbouring germ cells (*n*), degenerating cells showing nuclear pyknosis (arrowhead) in 20 mg/kg trazodone-administered rats. (a–d) High magnification of Leydig cells. (a) Normal morphological integrity of Leydig cells in control rats. (b) Slight vacuolation and deformation in Leydig cells (arrowhead) in 5 mg/kg trazodone-administered rats. (c) Intense vacuolation and deformation in Leydig cells (arrowhead) in 10 mg/kg trazodone-administered rats. (d) Complete deformation (arrowhead) and lysis (arrow) of Leydig cells in 20 mg/kg trazodone-administered rats.

Histological alterations as a result of TRZ administration at different doses are shown in Figures 3 and 4. Control group animals manifested regular feature of the seminiferous tubules, germinal epithelial cells, and interstitial cells (Figures 3 and 4).

5 mg/kg TRZ administration resulted in mild central degeneration of the tubules. Cells at the center of the tubules were separated from each other and some of them accumulated in the lumen. Thickening of basement membrane and vacuolation in Sertoli cells were also observed (Figures 3 and 4).

In 10 mg/kg TRZ-administered group, many of the germ cells abnormally accumulated in the lumen. Large vacuoles were observed in the germinal epithelium. Deformated Sertoli cells were partially detached from the basement membrane. Leydig cells showed intense vacuolation and deformation (Figures 3 and 4).

20 mg/kg TRZ administration resulted in total degeneration of the seminiferous tubules. Cells of adluminal compartment disintegrated and desquamated into the lumen. Architecture of germinal epithelium was disorganized. Necrosis was observed in Sertoli cells and neighboring germ cells. Degenerating cells were showing nuclear pyknosis. Lysis of the cytoplasm of Leydig cells was also observed (Figures 3 and 4). Johnsen's scores and semiquantitative comparison of pathology at the cellular level are shown in Table 3.

### 3.5. Effects of TRZ Treatment on the Serum Hormone Levels in Rats

When the groups were compared in terms of serum FSH levels, statistically significant dose-related increases were observed in the TRZ-administered groups compared to the control group. Compared to the control group, a statistically significant increase was found in the serum LH levels in the 10 and 20 mg/kg TRZ-administered groups. When the groups were compared in terms of serum testosterone levels, statistically significant increases were found in the TRZ-administered groups. No significant differences were obtained among the TRZ-administered groups in terms of serum FSH, LH, and testosterone levels (Table 4).

### 3.6. Effects of TRZ Treatment on GSH and MDA Levels in Testis Tissue of Rats

GSH levels of testis tissues did not show any significant difference in the TRZ-administered groups in comparison to the control group. No significant differences were observed among the TRZ-administered groups in terms of GSH levels in testis tissues. When the groups were compared in terms of the MDA levels of testis tissue, dose-related increases were observed in the TRZ-administered groups compared to the control group (Table 5).

## 4. Discussion

According to our study results, which we performed independently of other risk factors related to reproductive toxicity, TRZ administration decreased sperm concentration, motility, and normal sperm morphology, increased sperm DNA damage, and induced degeneration of testicular structure. Detected reproductive toxicity findings were accompanied by increases of serum FSH, LH, and testosterone levels and oxidative stress in the testicular tissue.

The reproductive function of men is evaluated via semen analysis, by assessing sperm concentration, motility, and morphology. These parameters provide information about sperm quality [61, 62]. Spermatozoa must be produced in sufficient numbers and exhibit normal motility and shape for normal sperm function [61]. It was noteworthy that sperm concentration, motility, and normal sperm morphology decreased in TRZ-administered groups dose-dependently. As known, pregnancy rates by intercourse and intrauterine insemination decline as sperm density decreases. The efficient passage of spermatozoa through cervical mucus is dependent on rapid progressive motility. Persistent poor motility is a good predictor of failure in fertilization [63]. Sperm morphology measurement still has a very important role in the clinical evaluation of sperm fertilization capacity [62]. In general, pregnancy is possible with low morphology scores and that both motility and morphology have also demonstrated prognostic value [63]. Otherwise, it is emphasized that tail anomalies are positively correlated with infertility and are an important parameter affecting motility [55, 64, 65]. When tail and head anomalies were evaluated in groups, tail anomalies were found to be higher than head anomalies in TRZ groups. These increased tail anomalies in TRZ groups may also reflect decreased sperm motility.

Recently, there has been a focus on the analysis of sperm DNA damage, as an indicator of sperm quality and fertility. The most common types of identified sperm DNA damage are single and double DNA strand breaks, the chemical modification of a base, inter- or intrastrand cross-linkage, and DNA-protein cross-links [62, 66]. Increased sperm DNA damage can cause a lower fertilization potential, a lower blastocyst formation rate, a lower implantation rate, and adverse effects on embryo development [67–69]. The limited repair capacity of sperm DNA makes it more susceptible to damage [70, 71]. The Comet method, which is often used as a reliable method for determining sperm DNA damage, is especially precise when determining DNA errors associated with double-strand breaks [72–75]. The tail moment calculated in this method is a parameter that is frequently used in terms of the comparability of DNA damage among groups. When the tail moment was compared between the groups, it was notable that DNA damage increased in TRZ-treated groups dose-dependently. Additionally, there are positive correlations between abnormal sperm morphology and sperm DNA damage, but not DNA fragmentation [3, 72]. At this point, previous findings supported our results, which showed that induced sperm abnormalities were accompanied by sperm DNA damage in TRZ-administered groups.

Histopathological analysis is frequently used as an important biomarker in toxicity research [76]. Reduced sperm quality and quantity can also be accompanied by histopathological changes in the testes [77]. The first pathological findings observed were slight vacuolization in seminiferous tubules and Leydig cell deformation in the low-dose TRZ group. While basal lamina irregularity, vacuolar enlargement, and Leydig cell deformation were increased in 10 mg/kg TRZ-administered group, testicular degenerative findings progressing to lysis and necrosis in the cells were notable in high-dose TRZ group.

The spermatogenesis process is regulated by endocrine activity of the hypothalamus-pituitary-testicular axis. FSH and LH are released from the anterior pituitary to maintain spermatogenesis. While LH mediates release of testosterone from Leydig cells, FSH mediates the release of androgen-binding protein from Sertoli cells which is required for sperm maturation [78, 79]. In our study, serum FSH, LH, and testosterone levels were increased in TRZ-administered groups dose-dependently. At this point, it could be stated that secondary to increased levels of LH, testosterone plasma levels increased in TRZ-administered groups. On the other hand, previous studies investigating the effects of serum hormone levels on sperm parameters demonstrated that FSH and LH levels showed negative correlation with sperm concentration, motility, and morphology [80–82], testosterone level did not affect sperm parameters or only positively correlated with motility [81–84]. In our study, significant reductions in sperm quality with TRZ administration were also correlated with increases in serum FSH and LH levels. Additionally, the degenerative findings identified in the testis tissues may also be associated with the increased serum LH levels as well as the oxidative stress induced by TRZ. Some studies have shown that increased LH levels cause degeneration of the germinal cells, which negatively affect spermatogenesis [85].

Oxidative stress, which occurs as a result of oxidant/antioxidant imbalance in favor of oxidants, can cause deteriorations in testicular structure and spermatogenesis process and, correspondingly, infertility [86–88]. Testis tissue is vulnerable and also highly dependent on oxygen to drive spermatogenesis process and highly susceptible to the toxic effects of reactive oxygen metabolites; in this context, the testis is very similar to the brain [86]. Furthermore, cell membranes of spermatozoa are rich in polyunsaturated fatty acids which makes them more susceptible to oxidative damage [89]. Animal models demonstrated a causal relationship between the induction of oxidative stress in the testes and the impairment of male reproductive function [86]. Oxidative stress is defined as either an excessive production of reactive oxygen species/reactive nitrogen species and/or a deficiency of enzymatic and nonenzymatic antioxidants in the biological system. We evaluated the oxidative status by measuring the level of MDA which is the end product of lipid peroxidation and the level of GSH which is an important nonenzymatic antioxidant in testis tissue [90]. In our study, MDA levels increased significantly and dose-dependently, but no significant difference was found in terms of GSH levels in TRZ-administered rats. According to these findings, it could be asserted that TRZ induced oxidative stress in the testicular tissue. A wide variety of different xenobiotics have also been shown to induce oxidative stress in the testes [86]. Toxicity studies indicated TRZ-induced hepatotoxicity via oxidative stress in isolated hepatocyte. Also, these studies emphasized that the bioactive intermediate metabolites of TRZ might cause hepatotoxicity [32, 33, 91]. These metabolites are the reactive quinoneimine and epoxide species formed by the cytochrome P4503A4 (32). Excessive extrinsic reactive metabolites cause plasma membrane damage and negatively affect sperm parameters such as sperm concentration, motility, and morphology [62, 71, 89, 92]. Otherwise, excessive intrinsic reactive metabolites cause DNA damage and induce sperm DNA strand breaks [62, 66, 71]. Additionally, ongoing oxidative stress in testicular tissue is known to cause histopathological changes [87, 93, 94]. Oxidative stress can induce testicular atrophy and degeneration of seminiferous tubules by disrupting membrane integrity [95]. In our study, decreased sperm quality, increased sperm DNA damage, and testicular degenerative findings could be the result of TRZ-induced testicular oxidative stress.

In conclusion, our study outlines the reproductive toxicity of TRZ, commonly used to treat depression and insomnia, in male rats with respect to certain reproductive parameters. TRZ-induced reproductive toxic effects may be a consequence of increased serotonin associated with this agent and/or direct toxic effects of the agent/metabolites in the reproductive system. We emphasize that clinical researches are very important in patients under TRZ treatment. Determination of sperm parameters in patients before, during, and after TRZ treatment will contribute to the identification of its reproductive toxicity in males.

## Figures and Tables

**Table 1:** Effects of TRZ on relative organ weights of male rats.

	C	TRZ-5	TRZ-10	TRZ-20
Relative left testis weights (g/100 g BW)	0.49 ± 0.01	0.51 ± 0.02	0.50 ± 0.01	0.50 ± 0.01
Relative left epididymis weights (g/100 g BW)	0.20 ± 0.004	0.20 ± 0.004	0.22 ± 0.004	0.19 ± 0.02

C: control group; TRZ-5: 5 mg kg^−1^ trazodone-treated rats for the 28-day group; TRZ-10: 10 mg kg^−1^ trazodone-treated rats for the 28-day group; TRZ-20: 20 mg kg^−1^ trazodone-treated rats for the 28-day group. All data were expressed as mean ± standard error.

**Table 2:** Effects of TRZ on the sperm concentration, motility, and morphology of male rats.

	C	TRZ-5	TRZ-10	TRZ-20
Sperm concentration (10^6^/ml)	4.68 ± 0.30	3.04 ± 0.21^∗^	2.84 ± 0.12^∗∗∗^	2.68 ± 0.21^∗∗∗^
Sperm motility (%)	86.49 ± 1.23	80.06 ± 0.93^∗^	78.85 ± 1.01^∗^	76.23 ± 0.83^∗∗∗^
Abnormal sperm count (%)	18.00 ± 1.12	28.90 ± 1.98^∗^	31.20 ± 0.65^∗∗∗^	37.08 ± 1.10^∗∗∗^

C: control group; TRZ-5: 5 mg kg^−1^ trazodone-treated rats for the 28-day group; TRZ-10: 10 mg kg^−1^ trazodone-treated rats for the 28-day group; TRZ-20: 20 mg kg^−1^ trazodone-treated rats for the 28-day group. All data were expressed as mean ± standard error. ^∗^Different from C (*p* < 0.05). ^∗∗∗^Different from C (*p* < 0.001).

**Table 3:** Johnsen's scores and semiquantitative comparison of pathology at the cellular level.

	Johnsen's score ± SE (*n* = 160)	Spermatogonial swelling	Cytoplasmic vacuolation	Deformation of cellular architecture
C	9.60 ± 0.45	−	−	−
TRZ-5	9.15 ± 0.24^∗^	+	+	−
TRZ-10	8.69 ± 0.29^∗^	++	++	++
TRZ-20	7.70 ± 0.34^∗^	+++	+++	+++

Johnsen's mean testicular biopsy scores (1: no cells–10: complete spermatogenesis) and three pathological viewpoints (spermatogonial swelling, cytoplasmic vacuolation, and deformation of cellular architecture) estimated at the cellular level on a semiquantitative scale according to their degrees (+: low; ++: moderate; +++: high). C: control group; TRZ-5: 5 mg kg^−1^ trazodone-treated rats for the 28-day group; TRZ-10: 10 mg kg^−1^ trazodone-treated rats for the 28-day group; TRZ-20: 20 mg kg^−1^ trazodone-treated rats for the 28-day group. All data were expressed as mean ± standard error. ^∗^Different from C (*p* < 0.05).

**Table 4:** Effects of TRZ on serum hormone levels of male rats.

	C	TRZ-5	TRZ-10	TRZ-20
FSH (IU/l)	32.50 ± 2.61	48.48 ± 2.34^∗^	58.54 ± 1.31^∗∗∗^	59.97 ± 4.00^∗^
LH (mg/dl)	5.77 ± 0.12	5.17 ± 0.39	9.07 ± 0.37^∗∗∗^	11.03 ± 0.78^∗∗∗^
Testosterone (IU/l)	2.53 ± 0.23	3.40 ± 0.16^∗^	3.68 ± 0.17^∗^	3.84 ± 0.12^∗^

C: control group; TRZ-5: 5 mg kg^−1^ trazodone-treated rats for the 28-day group; TRZ-10: 10 mg kg^−1^ trazodone-treated rats for the 28-day group; TRZ-20: 20 mg kg^−1^ trazodone-treated rats for the 28-day group. All data were expressed as mean ± standard error. ^∗^Different from C (*p* < 0.05). ^∗∗∗^Different from C (*p* < 0.001).

**Table 5:** Effects of TRZ on MDA and GSH levels in testis tissues of male rats.

	C	TRZ-5	TRZ-10	TRZ-20
MDA (pmol ml^−^)	424.03 ± 10.69	543.78 ± 8.09^∗∗∗^	590.58 ± 8.31^∗∗∗^	690.83 ± 11.08^∗∗∗^
GSH (*μ*M)	24.55 ± 1.86	25.18 ± 0.96	24.70 ± 1.24	25.20 ± 1.21

C: control group; TRZ-5: 5 mg kg^−1^ trazodone-treated rats for the 28-day group; TRZ-10: 10 mg kg^−1^ trazodone-treated rats for the 28-day group; TRZ-20: 20 mg kg^−1^ trazodone-treated rats for the 28-day group. All data were expressed as mean ± standard error. ^∗∗∗^Different from C (*p* < 0.001).
