# Corrigendum to “Toxic Effects of Trazodone on Male Reproductive System via Disrupting Hypothalamic-Pituitary-Testicular Axis and Inducing Testicular Oxidative Stress”

**DOI:** 10.1155/2018/8294061

**Published:** 2018-11-05

**Authors:** Sinem Ilgın, Gözde Aydoğan-Kılıç, Merve Baysal, Volkan Kılıç, Mina Ardıç, Şeyda Uçarcan, Özlem Atlı

**Affiliations:** ^1^Faculty of Pharmacy, Department of Pharmaceutical Toxicology, Anadolu University, Eskisehir, Turkey; ^2^Faculty of Science, Department of Biology, Anadolu University, Eskisehir, Turkey

In the article titled “Toxic Effects of Trazodone on Male Reproductive System via Disrupting Hypothalamic-Pituitary-Testicular Axis and Inducing Testicular Oxidative Stress” [1], there was a missing grant number. An acknowledgment should be added as follows:

“This study was supported by Anadolu University Scientific Research Projects Commission, (Grant Number: 1401S018)”.

## References

[1] Ilgın S., Aydoğan-Kılıç G., Baysal M. (2018). Toxic effects of trazodone on male reproductive system via disrupting hypothalamic-pituitary-testicular axis and inducing testicular oxidative stress. *Oxidative Medicine and Cellular Longevity*.

